# Evaluating a Modified Coblation Technique in Adenoidectomy: A Single‐Blind Randomized Study

**DOI:** 10.1002/oto2.70162

**Published:** 2025-09-23

**Authors:** Necdet Özçelik, Aslı Çakır, Elvin Alaskarov

**Affiliations:** ^1^ Department of Otorhinolaryngology Istanbul Medipol University Health Care Practice and Research Center Esenler Hospital Istanbul Turkey; ^2^ Department of Pathology Istanbul Medipol University Istanbul Turkey

**Keywords:** adenoidectomy, coblation, histopathology, pediatric surgery, saline irrigation, surgical innovation, thermal injury, wand clogging

## Abstract

**Objective:**

To evaluate the clinical and histopathological advantages of a modified technique—In Saline Coblation Adenoidectomy (ISCA)—over conventional coblation adenoidectomy (CCA) in pediatric patients, with respect to intraoperative efficiency, tissue preservation, and postoperative outcomes.

**Study Design:**

This study was designed as a prospective, randomized, single‐blind trial. Patients were randomly assigned to two groups, Group A “CCA” and Group B “ISCA,” each consisting of 25 children. Following the approval of the Medipol University Ethics Committee, patients who underwent adenoidectomy or adenotonsillectomy were included in the study.

**Setting:**

In Group A, adenoid tissue was ablated using the coblator's built‐in irrigation system. For Group B, the nasopharynx and, partially, the oral cavity were continuously filled with saline solution delivered via the nasal passage. Excess fluid was aspirated from the mouth using a dedicated suction tip. This ensured that the endoscope and coblator tip remained immersed in saline throughout the procedure.

**Methods:**

A prospective, randomized, single‐blind study was conducted involving 50 pediatric patients who underwent either conventional coblation (Group A, n = 25) or ISCA (Group B, n = 25). Operative time, intraoperative blood loss, postoperative pain scores, and wand‐related issues were recorded. Histopathological analysis of adenoid specimens was performed to assess tissue integrity and thermal injury. Patients were followed for 6 to 18 months postoperatively for recurrence and complications.

**Results:**

ISCA significantly reduced operative time compared to CCA (24 ± 5.8 minutes vs 33 ± 8.5 minutes; *P* < .05). Wand tip clogging and secondary wand use were observed only in Group A. Histopathological analysis revealed greater epithelial preservation and reduced carbonization in Group B (92% vs 0%; *P* < .001). Postoperative complications such as transient velopharyngeal insufficiency and localized infection occurred exclusively in Group A, whereas no statistically significant difference in recurrence or residual tissue was noted between the groups.

**Conclusion:**

The ISCA technique offers clear clinical advantages over conventional coblation by improving procedural efficiency, minimizing collateral thermal injury, and eliminating wand‐related delays. These findings support its wider adoption in high‐volume pediatric otolaryngology settings.

Adenoidectomy has remained one of the most frequently performed surgical procedures in pediatric otolaryngology. It is commonly indicated in cases of obstructive sleep apnea, otitis media with effusion, recurrent otitis media, persistent nasal obstruction, and chronic rhinosinusitis.^1^, ^2^

Despite considerable technological advancements that have improved the safety and precision of adenoid surgery, the procedure is not without risks. Potential complications—including postoperative hemorrhage, anesthesia‐related issues, aspiration, pulmonary edema, atlantoaxial subluxation, mandibular trauma, Eustachian tube injury, nasopharyngeal stenosis, and velopharyngeal insufficiency—remain of concern, particularly in young children.^3^, ^4^

The evolution of surgical technique in adenoidectomy has progressed from blind digital curettage to mirror‐guided and more recently to endoscopically assisted approaches, which have significantly enhanced surgical visualization and reduced the risk of incomplete resection.^5^ Moreover, the integration of newer energy‐based modalities—such as suction diathermy, microdebriders, lasers, and coblation—has allowed for more controlled and targeted tissue removal.

Coblation technology, in particular, has gained popularity due to its ability to ablate tissue at relatively low temperatures, theoretically minimizing collateral thermal damage.^6^, ^7^

However, conventional coblation adenoidectomy (CCA) is not without intraoperative challenges. Surgeons often encounter issues such as wand tip clogging and inadequate cooling at the surgical site, especially when the operative field is poorly hydrated.^8^, ^9^ These factors may increase operative time, reduce surgical efficiency, and contribute to increased equipment costs due to the need for secondary wands.

To address these limitations, we introduced a novel technical modification: In Saline Coblation Adenoidectomy (ISCA).

The aim of this study was to statistically compare the intraoperative and postoperative outcomes of CCA and the ISCA technique. By evaluating metrics such as operative time, blood loss, wand usage, postoperative pain, tissue histology, and complication rates, we aimed to determine whether this saline‐assisted modification offers tangible clinical and technical advantages in pediatric adenoid surgery.

## Materials and Methods

### Study Design and Patient Selection

This study was designed as a prospective, randomized, single‐blind trial. Patients were randomly assigned to two groups, each consisting of 25 children. Following the approval of the Medipol University Ethics Committee, patients who underwent adenoidectomy or adenotonsillectomy were included in the study (Date: 14.09.2022, Decision No. E‐10840098‐202.3.02‐5287).

Routine preoperative surgical and anesthetic assessments were performed for all patients. Demographic data and clinical evaluations were recorded before surgery.

During preoperative flexible endoscopic evaluation, the degree of choanal obstruction caused by adenoidal tissue was graded using the McMurray and Clemens classification system (Grades I‐IV). Grade I was defined as adenoid tissue filling one‐third of the choanal vertical height; Grade II, two‐thirds; Grade III, nearly complete but not total obstruction; and Grade IV, complete choanal blockage.^10^

For patients who could not tolerate flexible endoscopy due to poor cooperation, adenoid size was assessed using lateral nasopharyngeal radiographs.

Operative time was measured from the insertion of a pediatric tongue depressor with a Davis mouth gag to the completion of adenoid tissue removal. In cases where tonsillectomy was also performed, this duration was not included in the recorded operative time.

Intraoperative blood loss was quantified by subtracting the amount of saline used for irrigation from the total volume of fluid collected in the suction canister.

Following the surgery, the operative site in the nasopharynx was evaluated in all patients. In both groups, a punch biopsy specimen was obtained immediately after the completion of ablation, before final suctioning. The specimen was taken from the central surgical site, adjacent to the ablated area, to assess the degree of peripheral thermal spread without sampling overtly charred or nondiagnostic tissue. This method was applied consistently across both groups.

Patients were followed up for 1.5 years postoperatively. Symptoms were compared to preoperative complaints and classified as resolved, improved, unchanged, or worsened. Additionally, the nasopharyngeal region was examined for signs of recurrence.

### Surgical Technique

All procedures were performed under general anesthesia with orotracheal intubation by the same surgical team. In both the coblation groups, a 4‐mm 30‐degree rigid endoscope was used transorally throughout the procedure to visualize the nasopharynx during adenoidectomy. This ensured continuous and direct monitoring of the surgical field. The Procise XP Wand system (ArthroCare Corp) was utilized for tissue ablation and coagulation. Power settings were adjusted according to the tissue volume: 7 to 8 for ablation and 3 for coagulation.

### Group A: CCA

In Group A (Conventional Coblation), the saline was delivered through the standard coblation system, which provides saline to the wand tip upon foot pedal activation. To ensure consistent flow, the flow regulator on the infusion set was fully opened for all cases, allowing unrestricted saline delivery during activation. In cases where the nasopharynx was inadequately filled with fluid, wand tip clogging occasionally necessitated the use of an additional wand.

### Group B: ISCA

In Group B (ISCA) (Supplemental Video S1, available online), in addition to the same coblator‐integrated irrigation system used in Group A, a separate continuous saline stream was established. This was achieved by passing a sterile saline tube transnasally into the nasopharynx to deliver free‐flowing saline directly to the surgical field. The purpose of this additional irrigation was to maintain a stable fluid environment within the nasopharynx, allowing both the coblation wand tip and the endoscope to remain submerged in saline throughout the procedure. This setup reduced thermal spread and minimized wand clogging, contributing to the improved operative efficiency observed in Group B. Excess fluid in the oropharynx was continuously evacuated using a malleable suction tip, commonly used in dental procedures, positioned at the corner of the mouth.

In the ISCA group, the additional saline irrigation was administered using a standard intravenous infusion set, fully opened to allow free‐flow of saline through a nasal catheter positioned into the nasopharynx. The continuous saline irrigation was administered via a nasal catheter positioned before the start of ablation. The process was initiated and monitored by an assisting surgical nurse or resident, but it did not require constant manual control or an additional set of hands during the procedure. Excess fluid accumulating in the oropharynx was simultaneously removed using a malleable dental suction tip placed at the corner of the mouth.

**Figure 1:**
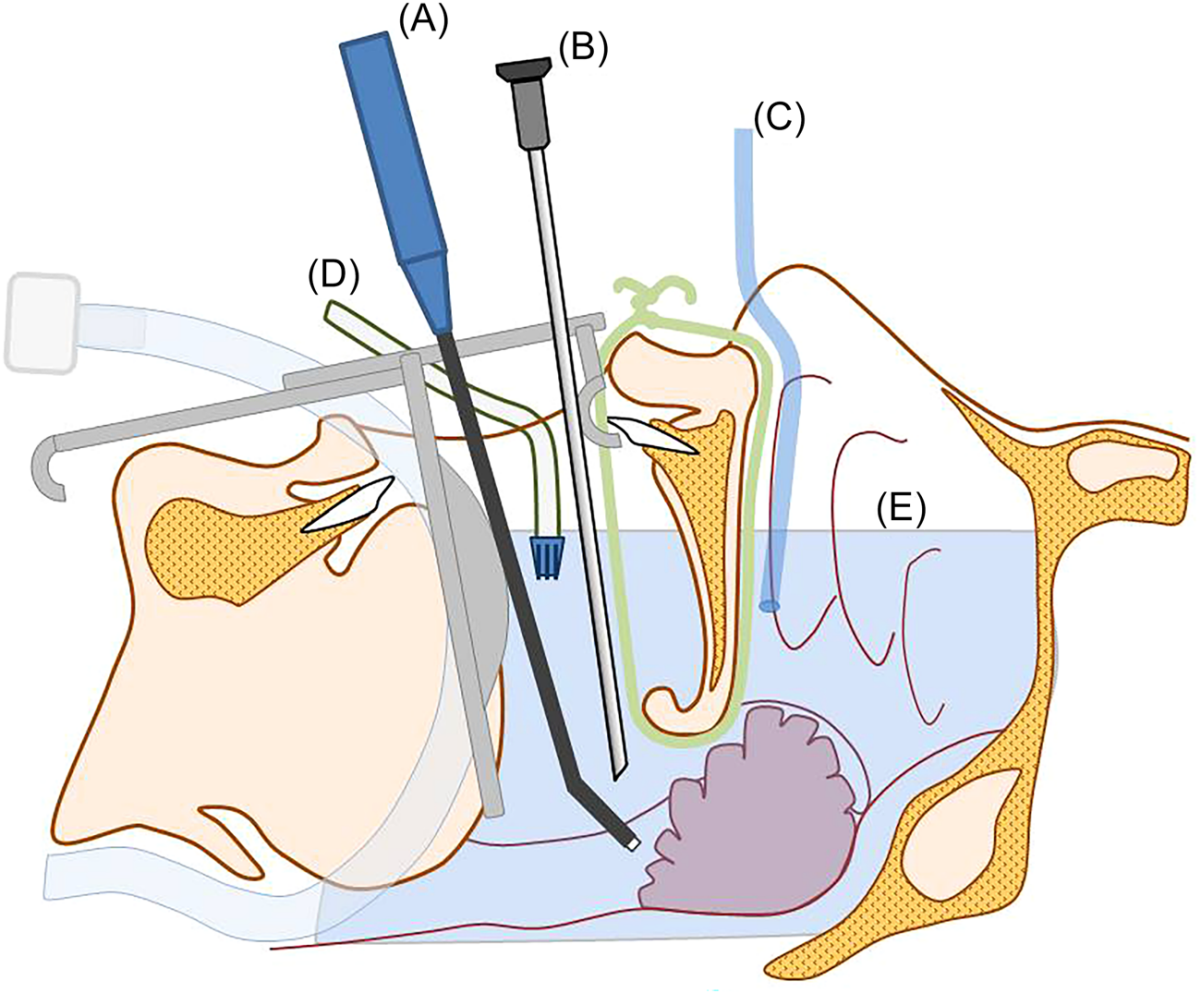
Schematic representation of the surgical field demonstrating the In Saline Coblation Adenoidectomy technique. (A) Coblator device, (B) 30‐degree endoscope, (C) continuous saline flow, (D) suction tip removing excess fluid from the oral cavity, and (E) saline level maintained at a constant height.

This technique ensured that both the endoscope and the coblator wand remained consistently immersed in a stable saline environment throughout the procedure. Importantly, this system was self‐sustaining and did not require a second operator. The continuous saline flow not only improved visualization (by preventing blood‐induced blurring) but also minimized thermal injury, as confirmed by reduced cellular damage at the resection margins (Figure 1). The illustration highlights the thermal stability and tissue‐preserving effect created by continuous saline flow within the nasopharynx.

## Results

A total of 50 pediatric patients were enrolled in the study, with 25 patients in each group: Group A (CCA) and Group B (ISCA).

The demographic and baseline clinical characteristics were comparable between the two groups (Table 1). The mean age was 5.5 ± 2.2 years in Group A and 5.8 ± 2.5 years in Group B (*P* = .675). There was no statistically significant difference in sex distribution (*P* = .158), adenoid size (Group A: 3.29 ± 0.46 vs Group B: 3.33 ± 0.50; *P* = .590), or the type of surgery performed (adenoidectomy vs adenotonsillectomy; *P* = .452).

**Table 1:** Demographic and Clinical Characteristics of Each Group of Patients

	Group A CCA (n = 25)	Group B ISCA (n = 25)	*P* value^a^
Sex, n (%)			
Male	12 (48)	15 (60)	.158^a^
Female	13 (52)	10 (40)	
Age, y, mean ± SD	5.5 ± 2.2	5.8 ± 2.5	.675^b^
Adenoid grade (±SD)	3.29 (±0.46)	3.33 (±0.50)	.590^b^
Surgery preformed, n			
Adenoidectomy	19	20	.452^a^
Adenotonsillectomy	6	5	

Abbreviations: CCA, conventional coblation adenoidectomy; ISCA, In Saline Coblation Adeniodectomy; SD, standard deviation.

^a^ Chi‐square test.

^b^ Two‐sample *t* test.

Intraoperative and postoperative findings are summarized in Table 2. The mean operative time was significantly shorter in Group B compared to Group A (24 ± 5.8 minutes vs 33 ± 8.5 minutes, respectively; *P* < .05). Intraoperative blood loss was comparable between the groups (12 ± 2.3 mL in Group A vs 11 ± 2.7 mL in Group B; *P* = .30). Postoperative pain scores also revealed no statistically significant difference (Group A: 4.02 ± 0.79 vs Group B: 3.85 ± 0.45; *P* = .25).

**Table 2:** Comparison of the Intraoperative and Postoperative Parameters of Each Group of Patients

	Group A CCA (n = 25)	Group B ISCA (n = 25)	*P* value^a^
Mean operative time, min (±SD)	33 ± 8.5	24 ± 5.8	**<.05**
Intraoperative blood loss, mL (±SD)	12 ± 2.3	11 ± 2.7	.30
Postoperative pain score (±SD)	4.02 (±0.79)	3.85 (±0.45)	.25

Values that are statistically significant are presented in bold in the Table.

Abbreviations: CCA, conventional coblation adenoidectomy; ISCA, In Saline Coblation Adeniodectomy; SD, standard deviation.

^a^ Mann‐Whitney test.

Wand‐related intraoperative issues were significantly more prevalent in Group A. Wand clogging occurred in 24% of patients in Group A, whereas no instances were reported in Group B. Additionally, secondary wand replacement was necessary for 16% of patients in Group A, but none of the ISCA cases required this intervention. The mean wand‐related time delay was 4.3 ± 1.2 minutes in Group A, compared to zero minutes in Group B (Table 3).

**Table 3:** Intraoperative Wand Usage in Both Groups

Parameter	Group A (CCA)	Group B (ISCA)
Wand clogging incidence	6/25 (24%)	0/25 (0%)
Secondary wand requirement	4/25 (16%)	0/25 (0%)
Mean wand‐related time delay, min	4.3 ± 1.2	0

Abbreviations: CCA, conventional coblation adenoidectomy; ISCA, In Saline Coblation Adeniodectomy.

**Figure 2:**
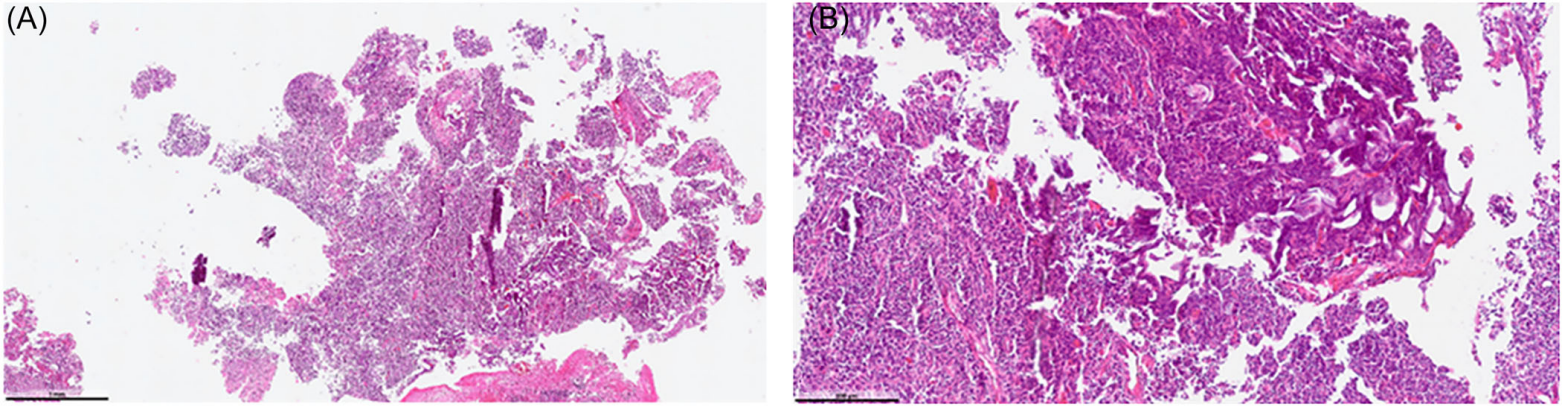
Histopathological evaluation of punch biopsy from the surgical site in the conventional coblation adenoidectomy group following coblation.

Histopathological examination of resected specimens demonstrated more extensive thermal injury in Group A, including coagulative necrosis, carbonization, and epithelial disruption (Figure 2). (2A) Lymphoid tissue exhibiting fragmentation and sectioning artifacts, with stromal disruption and areas where overall tissue integrity is not clearly preserved (hematoxylin and eosin [H&E], ×40). (2B) At higher magnification, prominent crushing and shrinkage artifacts are observed, along with regions of diminished cellular detail (H&E, ×200).

**Figure 3:**
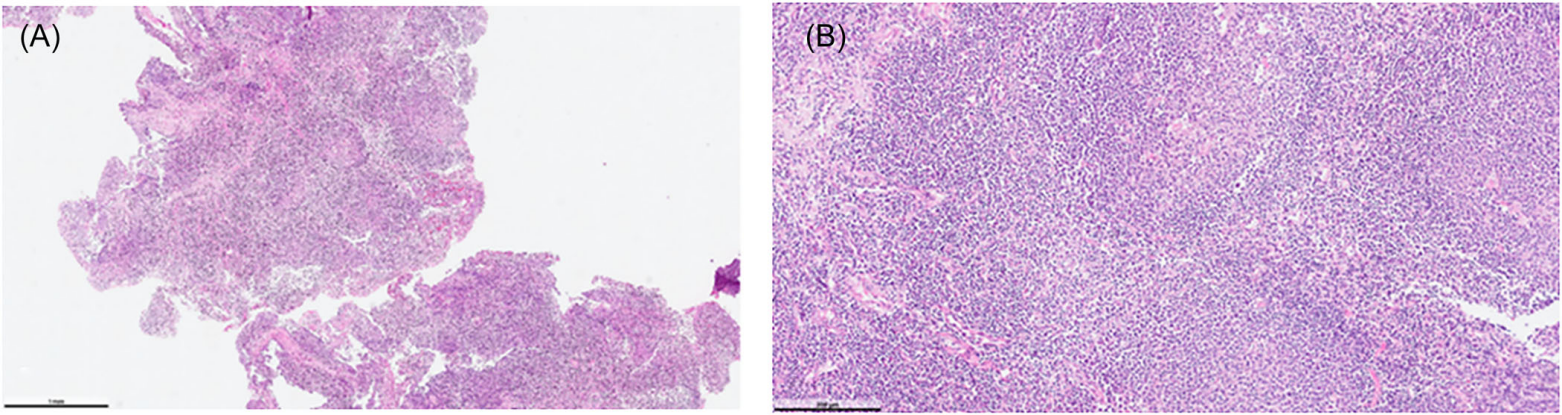
Histopathological evaluation of punch biopsy from the surgical site in the In Saline Coblation Adenoidectomy group following coblation.

In contrast, Group B showed better preservation of epithelial integrity, with minimal stromal edema and absence of carbonization in 92% (23/25) of samples (*P* < .001). These findings support the notion that the saline environment in ISCA provides a thermal buffer effect that minimizes collateral tissue damage (Figure 3). (3A) Lymphoid tissue with optimal sectioning and staining quality, showing well‐preserved germinal centers and paracortical areas (H&E, ×40). (3B) At higher magnification, the morphological details of lymphoid and associated cellular components are clearly distinguishable (H&E, ×200).

Postoperative endoscopic assessments at multiple time points (10 days, 1 month, 3 months, and 6 months) revealed no significant differences between the groups regarding residual adenoid tissue or choanal patency. These findings suggest equivalent efficacy in adenoid removal between the two techniques.

In Group A, one patient was rehospitalized on postoperative day 4 due to high fever and halitosis. Endoscopic examination of the nasopharynx revealed purulent discharge and necrosis at the surgical site. Nasopharyngeal secretions were aspirated, and nasal irrigation was performed. Laboratory tests showed elevated infection markers, prompting the initiation of intravenous antibiotic therapy with ceftriaxone (500 mg twice daily). By the third day of treatment, the patient's symptoms and clinical condition had resolved, and the patient was discharged.

In both groups, one patient each presented with minor postoperative bleeding characterized by blood‐stained saliva during the first postoperative week. Endoscopic evaluation revealed a small, inactive bleeding site on the lateral wall of the nasopharynx. Conservative follow‐up without intervention was recommended in both cases.

In Group A, two patients developed transient velopharyngeal insufficiency within the first postoperative week, presenting with nasal speech and reduced speech intelligibility. On examination, edema of the soft palate and uvula was observed. Symptoms completely resolved by the second postoperative week without the need for further intervention.

In Group A, one patient presented with intermittent snoring and mouth breathing during sleep in the 10th postoperative month. Endoscopic follow‐up revealed Grade 2 adenoid regrowth around the choanae bilaterally. The patient was managed conservatively with anti‐allergic medical therapy and scheduled for ongoing observation (Table 4).

**Table 4:** Comparison of Complications According to Treatment Group

	Group A CCA (n = 25)	Group B ISCA (n = 25)
Infection	1	0
Postoperative bleeding	1	1
Velopharyngeal insufficiency	2	0
Rehospitalization	1	0
Recurrence	1	0

Abbreviations: CCA, conventional coblation adenoidectomy; ISCA, In Saline Coblation Adeniodectomy.

## Discussion

Coblation adenoidectomy has increasingly supplanted traditional cold curettage and electrocautery methods in pediatric adenoid surgery due to its ability to ablate tissue at lower temperatures, thereby reducing collateral thermal injury.^8^, ^11^ In our study, all patients in both groups underwent coblation adenoidectomy using the same device, with the coblator employed for both lymphoid tissue ablation and intraoperative hemostasis.

Despite these advantages, CCA is not without limitations. Commonly reported challenges include wand tip clogging, excessive thermal spread at the resection margins, and occasional interruptions in surgical workflow—issues that have been previously highlighted in comparative and technical analyses.^12^, ^13^

This study sought to address these issues by evaluating a novel approach—ISCA—in which the nasopharynx is continuously irrigated with saline during the procedure to improve cooling, visualization, and debris removal.

One of the most frequently cited disadvantages of coblation adenoidectomy compared to other techniques is its cost. A major contributor to this increased cost is the requirement for a single‐use coblation wand, which must be discarded after each case. Additionally, intraoperative malfunction or clogging of the wand—necessitating replacement—further increases procedural expenses.^13^, ^14^

In our series, secondary wand usage was observed in 4 out of 25 patients (16%) in the conventional coblation group. This highlights one of the key advantages of the ISCA technique, which entirely eliminated the need for a second wand.

Previous studies have also reported that coblation adenoidectomy may take longer than conventional curettage techniques due to device handling and visualization challenges.^9^, ^15^, ^16^ However, our findings demonstrated that ISCA significantly reduced operative time compared to the conventional coblation technique (24 ± 5.8 minutes vs 33 ± 8.5 minutes, *P* < .05). This difference can be primarily attributed to the higher incidence of wand clogging and the associated need for wand replacement in the conventional coblation group.

Compared to electrocautery or other thermal techniques, coblation generates lower tissue temperatures (60°C‐70°C), which results in less thermal injury at the surgical site and contributes to faster postoperative healing.^17^ Studies have also suggested that mucociliary activity is better preserved following coblation adenoidectomy than with traditional cold curettage methods.^18^

In our study, histopathological analysis of biopsy specimens from the surgical site revealed markedly less tissue necrosis in the ISCA group compared to the conventional group. In fact, the only case of postoperative fever (39°C) observed in the conventional group on postoperative day 4 was presumed to be associated with localized necrosis and secondary infection.

Moreover, two cases of transient velopharyngeal insufficiency in the conventional coblation group were associated with notable edema of the uvula and surrounding soft palate. This finding may be attributable to thermal diffusion from the wand tip in the absence of adequate local cooling. In contrast, in the ISCA group, the wand tip remained continuously surrounded by isotonic saline, which we believe helped limit lateral heat spread and protect surrounding tissues.

Further large‐scale studies are warranted to statistically validate differences in postoperative complications and to explore the long‐term clinical implications of reduced thermal injury with the ISCA technique.

Nonetheless, this study has several limitations. It was conducted at a single center with a relatively small sample size, which may limit generalizability. Although our histological findings were consistent and clinically relevant, a more quantitative scoring system could improve the objectivity of tissue preservation assessments. Future multicenter trials with larger patient populations and long‐term follow‐up are warranted to further validate the clinical and economic benefits of the ISCA technique.

In conclusion, ISCA represents a meaningful advancement in the surgical management of pediatric adenoid hypertrophy. By eliminating wand clogging, reducing operative time, preserving tissue integrity, and enhancing procedural efficiency, ISCA offers significant advantages over the conventional coblation technique. These findings support the broader adoption of ISCA, particularly in high‐volume surgical settings seeking to optimize outcomes while minimizing cost and complication risk.

## Authors' Note

Consent for Publication: The patients consented to the publication of this research.

## Author Contributions

**Necdet Özçelik**, Conception and design of the study, data acquisition, statistical analysis, manuscript drafting, data analysis and interpretation, critical revision of the manuscript, and final approval; **Aslı Çakır**, Statistical analysis, data analysis, and interpretation design of the study; **Elvin Alaskarov**, Conception and design of the study, manuscript drafting, interpretation, and critical revision.

## Disclosures

### Competing interests

The authors declare no conflicts of interest.

### Funding source

The authors have no funding or financial relationships to declare.

## Supporting information


**Supplemental Video S1:** Group B—In Saline Coblation Adenoidectomy (ISCA): intraoperative video footage.
